# Correction: Genetic characterization of the highlander Tibetan population from Qinghai-Tibet Plateau revealed by X chromosomal STRs

**DOI:** 10.1371/journal.pone.0278494

**Published:** 2022-11-28

**Authors:** The PLOS ONE Editors

## Notice of republication

This article was republished on November 17, 2022, to address a file issue. The article’s Data Availability statement has also been updated. Please download this article again to view the correct version.
